# An Innovative Optical Sensor for the Online Monitoring and Control of Biomass Concentration in a Membrane Bioreactor System for Lactic Acid Production

**DOI:** 10.3390/s16030411

**Published:** 2016-03-21

**Authors:** Rong Fan, Mehrdad Ebrahimi, Hendrich Quitmann, Matthias Aden, Peter Czermak

**Affiliations:** 1 Institute of Bioprocess Engineering and Membrane Technology, University of Applied Sciences Mittelhessen, Wiesenstr. 14, 35390 Giessen, Germany; rong.fan@lse.thm.de (R.F.); mehrdad.ebrahimi@lse.thm.de (M.E.); Hendrich.quitmann@ime.fraunhofer.de (H.Q.); 2 FAUDI Aviation GmbH, Scharnhorststr. 7B, 35260 Stadtallendorf, Germany; m.aden@faudi-aviation.com; 3 Department of Chemical Engineering, Kansas State University, 1005 Durland Hall, Manhattan, KS 66506, USA; 4 Faculty of Biology and Chemistry, Justus Liebig University Giessen, Heinrich-Buff-Ring 17, 35392 Giessen, Germany

**Keywords:** biomass measurement, online monitoring, optical sensor, lactic acid production, membrane filtration

## Abstract

Accurate real-time process control is necessary to increase process efficiency, and optical sensors offer a competitive solution because they provide diverse system information in a noninvasive manner. We used an innovative scattered light sensor for the online monitoring of biomass during lactic acid production in a membrane bioreactor system because biomass determines productivity in this type of process. The upper limit of the measurement range in fermentation broth containing *Bacillus coagulans* was ~2.2 g·L^−1^. The specific cell growth rate (µ) during the exponential phase was calculated using data representing the linear range (cell density ≤ 0.5 g·L^−1^). The results were consistently and reproducibly more accurate than offline measurements of optical density and cell dry weight, because more data were gathered in real-time over a shorter duration. Furthermore, µ_max_ was measured under different filtration conditions (transmembrane pressure 0.3–1.2 bar, crossflow velocity 0.5–1.5 m·s^−1^), showing that energy input had no significant impact on cell growth. Cell density was monitored using the sensor during filtration and was maintained at a constant level by feeding with glucose according to the fermentation kinetics. Our novel sensor is therefore suitable for integration into control strategies for continuous fermentation in membrane bioreactor systems.

## 1. Introduction

Bioprocess control is integral to modern biotechnology because it can improve reproducibility and system stability while reducing operating costs. Real-time process monitoring using sensor techniques facilitates bioprocess control by providing system information during the process, which can be used to regulate the corresponding system output. Over the last two decades, sophisticated sensors have been developed for upstream and downstream bioprocesses to enhance process efficiency and product quality. For example, many electrical, electrochemical and electromagnetic sensors have been commercialized for use in laboratory and industrial settings to measure pressure, temperature, pH and dissolved oxygen. 

In addition to conventional sensors, optical sensors are competitive candidates for bioprocess control. Diverse optical principles have been used to measure different physiochemical variables, including the measurement of vitamins, coenzymes [1] and pyruvate [2] by fluorescence spectroscopy, the determination of glucose concentrations by near-infrared spectroscopy [3,4], and the observation of cell morphology by *in situ* microscopy [5,6]. Light scattering can also be used to determine the density of many different microorganisms with high sensitivity [7]. In contrast to conventional offline measurement, online bioprocess monitoring using optical sensors is a noninvasive and nondestructive approach that does not interfere with cell metabolism and does not require periodic sampling, thus reducing the risk of contamination [8,9]. However, optical sensors must meet the specific requirements for each process, e.g., target parameters, data frequency, calibration, linearity, measurement range and longevity. Optical sensors must also overcome challenges specific to individual processes, such as the fouling of optical surfaces by the adhesion of microorganisms and/or proteins, and interference caused by medium components or gas bubbles. The appropriate sensor must be selected and characterized according to the process to ensure accurate measurement.

Lactic acid (LA) is a versatile carboxylic acid used in food products, beverages, cosmetics, medicines, and in the production of biodegradable polymers to replace petroleum-derived plastics. LA is commercially produced by the microbial conversion of carbohydrates such as dextrose, lactose and sucrose. Kinetic studies have shown that LA fermentation is a partially growth-associated process, in which both cell density and growth rate determine productivity [10,11,12]. However, LA fermentation is also a product-inhibited process. The accumulation of LA in batch or fed-batch processes inhibits cell growth and substrate conversion [13,14]. A membrane filtration device is therefore used in conventional batch or fed-batch processes to continuously remove LA from the reaction system, while the microorganisms remain in the reactor. Productivity is thus enhanced by increasing the biomass concentration and reducing product inhibition [15,16,17]. However, previous studies have shown that high cell densities cause severe membrane fouling, reducing the efficiency of the membrane during fermentation and necessitating regular membrane cleaning, which increases operational costs [18]. The biomass concentration must therefore be monitored and controlled within an appropriate range to balance the requirements of fermentation and filtration.

In membrane bioreactor (MBR) systems, crossflow filtration using microfiltration and/or ultrafiltration membranes is necessary to achieve cell retention. The membrane efficiency, *i.e.*, the permeate flow, is controlled predominantly by transmembrane pressure (TMP) and crossflow velocity (CFV). TMP is the driving force during filtration, and higher TMPs therefore enhance the permeate flow. However, excessive TMP leads to the formation of a gel layer or compact filtration cake on the membrane, which increases the hydraulic resistance during filtration. In contrast, the CFV helps to remove the cake layer from the membrane surface to maintain a stable permeate flow. Despite these positive effects, increasing the TMP or CFV also increases the mechanical energy input and thus the shear force, which may reduce cell vitality and growth. Therefore, when the boundary conditions for filtration are determined, both the filtration efficiency and the impact on cell growth must be considered.

Ideally, it would be possible to achieve the reliable and convenient measurement of microbial biomass *in situ*, and many different optical sensors have been developed to address this challenge. Here we describe an innovative scattered light sensor (AFGUARD^®^) which was used to monitor the biomass in a MBR system for the production of LA. The new sensor was compared to the optical density (OD) and cell dry weight (CDW) reference methods for the measurement of cell growth, allowing the statistical analysis of sensitivity and reproducibility. The impact of filtration parameters (TMP and CFV) on cell growth was characterized by measuring the maximum specific growth rate using the new optical sensor to evaluate its performance in practice. Furthermore, the biomass concentration was monitored and controlled during filtration to allow the characterization of ceramic ultrafiltration membranes.

## 2. Materials and Methods

### 2.1. Optical Sensor

**Figure 1:**
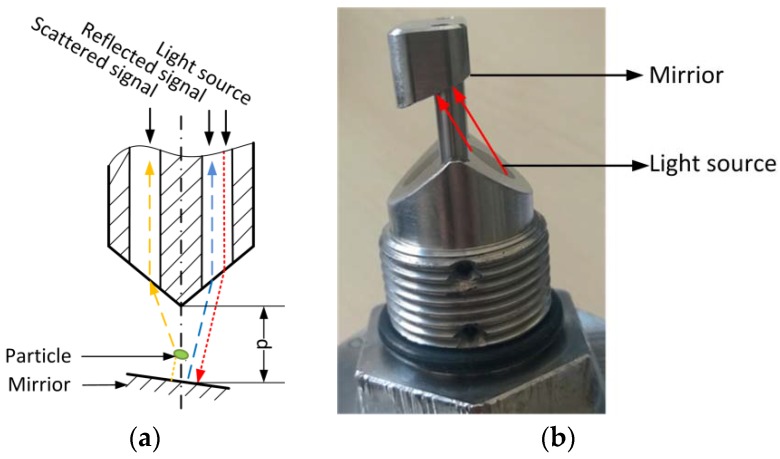
The AFG sensor, showing: (**a**) the measurement principle, d refers to the distance between the sensor and the mirror and (**b**) the device with an adjustable mirror.

The modified AFGUARD^®^ optical sensor, which was kindly provided by FAUDI Aviation GmbH (Stadtallendorf, Germany), was used to measure cell biomass during LA fermentation, and to monitor the filtration of the fermentation broth. The AFG sensor is an online monitoring device based on scattered light, which is designed for the *in situ* measurement of particulate matter. The measurement principle is shown in Figure 1a. The infrared light is reflected by a mirror and scattered by particles, whose concentration is determined by comparing the reflected and scattered light intensity. The measurement range can be adjusted by varying the distance between the mirror and the light source (Figure 1b). In our experiments, the mirror was removed to achieve the broadest measurement range. In this case, the sensor output only represents the intensity of scattered light detected by the receive channel.

### 2.2. Microorganisms and Fermentation Broth

*Bacillus coagulans* PS5, a facultative anaerobic homofermentative L(+) lactic acid-producing bacterium, was kindly supplied by Thyssenkrupp Industrial Solutions AG, Process Technologies, Leuna, Germany. The culture medium was modified MRS (de Man, Rogosa and Sharpe) medium, which contained 4.0 g·L^−1^ yeast extract, 8.0 g·L^−1^ meat extract, 10.0 g·L^−1^ peptone from casein, 2 g·L^−1^ K_2_HPO_4_, 0.1 g·L^−1^ MgSO_4_, 0.03 g·L^−1^ MnSO_4_, 1.0 g·L^−1^ Tween-80, and 20 g·L^−1^ glucose. The inoculum was grown in the same medium. All components were sterilized at 121 °C for 20 min except for glucose, which was sterilized separately. 

### 2.3. Ceramic Membranes

Different ceramic membranes were used for cell retention and LA recovery. The tubular ceramic α-Al_2_O_3_ membranes (19 channels) (Atech Innovations GmbH, Gladbeck, Germany) had a nominal pore size of 50 nm, an inner channel of 3.3 mm and an outer diameter of 25.4 mm. The effective membrane surface was ~0.09 m^2^. The ceramic hollow fiber membrane (31 fibers) (Mann + Hummel GmbH, Ludwigsburg, Germany) had a nominal pore size of 40 nm, an inner channel of 1.5 mm and an outer diameter of 25.4 mm. The effective membrane surface was ~0.031 m^2^.

### 2.4. Experimental Equipment

**Figure 2:**
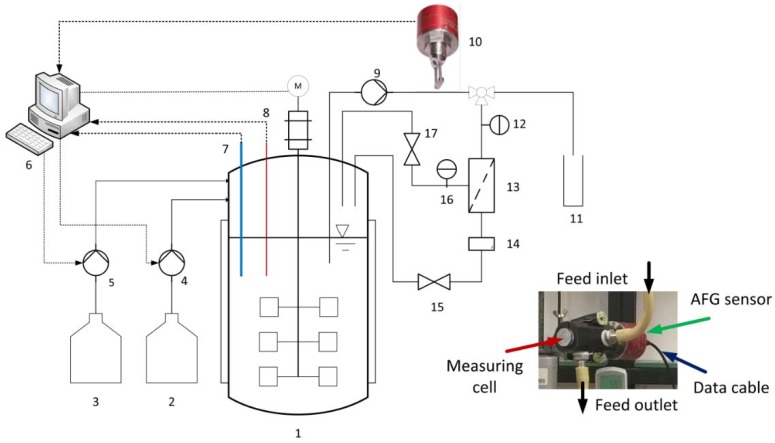
Dynamic measurement system for the characterization of the optical sensor and ceramic membranes: (**1**) stirred tank reactor, (**2**) base, (**3**) substrate, (**4**,**5**) peristaltic pumps, (**6**) control system, (**7**) pH probe, (**8**) temperature probe, (**9**) rotary vane pump, (**10**) optical sensor, (**11**) sample, (**12**,**16**) manometers, (**13**) membrane module, (**14**) miniature oval gear flow meter, (**15**,**17**) valves.

The configuration of the experimental equipment is shown in Figure 2. This system was used to test the ability of the AFG sensor and determine the membrane efficiency. The fermentations were carried out in a 1.5-L Biostat^®^ B stirred bioreactor (Sartorius Stedim Biotech GmbH, Göttingen, Germany) at 53 °C with neutralization achieved by adding 5 M NaOH. The fermentation broth was pumped through the membrane module and the measuring cell of the optical sensor using a rotary vane pump. The biomass concentrations were therefore detected by the AFG sensor in real time. The system was set to one of two operational configurations: first, when the permeate was closed, the correlation between the biomass concentration and the sensor readout was determined in batch fermentation mode with a bypass; second, when the permeate was open, the crossflow filtrations of the fermentation broth were carried out at different TMPs and CFVs in total recycle mode to determine the influences of mechanical energy input on the cell growth and the impacts of biomass concentration on the membrane efficiency. Here, the permeate and retentate were returned to the fermenter in order to maintain the concentration and temperature of the feed solution. The TMP and CFV were controlled by the rotary vane pump and a stainless steel valve, mounted on the retentate outlet. Permeate flow was monitored online with a miniature oval gear flow meter (B.I.O-Tech e.K., Vilshofen, Germany), and was then recycled back to the reactor. The membrane flux was calculated according to Equation (1) once the experiment was completed:

(1) $$J=\frac{F}{A}$$

where F refers to the volumetric flow rate of the permeate, and A is the membrane effective area. 

The TMP was varied between 0.2 and 1.2 bar, and the CFV was varied between 0.3 and 1.2 m·s^−1^. Because the permeate side was open during the experiments, the pressure on the permeate side was equal to atmospheric pressure. Therefore, the TMP was measured and monitored using two manometers located on the inlet (*P_1_*) and outlet (*P_2_*) of the membrane module, and was calculated using Equation (2):

(2) $$TMP=\frac{P_{1}+P_{2}}{2}$$

The CFVs (m·s^−1^) were calculated using the quotient of the retentate volumetric flow rate per inner cross-sectional area of the membrane tube. After each run, the system was rinsed with alkaline 1% Asiral^®^ (Asiral Industrie-Reiniger GmbH, Neustadt, Germany) at 53 °C for 1 h.

### 2.5. Analytical Methods

Glucose and lactate concentrations were measured using Biosen C_line GP (EKF-diagnostic GmbH, Barleben, Germany). The OD was measured using a UV/Vis spectrophotometer (Eppendorf AG, Hamburg, Germany). An aliquot was diluted with reverse osmosis (RO) water to ensure the absorbance was <0.5. The optical density (OD) of the dilution was then measured three times at 600 nm (OD_600_). To determine the CDW, 10-mL aliquots of cells were collected by centrifugation (10,000 rcf, 10 min, 4 °C) and transferred to a dried, pre-weighed 2-mL centrifuge tube. The cells were then resuspended in RO water and centrifuged at 16,100 rcf for 2 min at 4 °C (Eppendorf AG). The pellets were washed, centrifuged twice as above and dried at 55 °C until a constant mass was obtained.

### 2.6. Calculation

The specific growth rate (µ) was measured using the AFG sensor, OD_600_ values and CDW, and was used to characterize the kinetics of the LA fermentation. The data obtained from the AFG sensor were fitted to Equation (3) to calculate the maximum specific growth rate (µ_max_), where *N* refers to data captured during the exponential phase. *e* and *t* refer to the mathematical constant and the duration of the exponential phase. *N_0_* is a calculating constant to represent the sensor readout at the beginning of exponential phase, which is estimated by fitting the correlation between *N* and *t*.

(3) $$N=N_{0}\cdot e^{µ\cdot t}$$

For offline measurement, µ was defined as the increase in biomass per unit time (Equation (4)), where *M* is the OD_600_ value or CDW, as appropriate. *t_1_* and *t_2_* refer to the start and end of exponential phase.

(4) $$µ=\frac{\ln\frac{M_{t2}}{M_{t1}}}{t_{2}-t_{1}}$$

The mechanical energy input supplied by the rotary vane pump was characterized according to the pressure differential, shear stress (*τ*) and shear rate ($\dot{\gamma }$). The shear stress and the shear rate of fluid in the pipe were calculated using Equations (5) and (6). *η*, *v* and *d* are the dynamic viscosity, tangential flow velocity and inner diameter of membrane tubes channels.

(5) $$\tau =\frac{8\cdot \eta \cdot v}{d}$$

(6) $$\dot{\gamma }=\frac{8v}{d}$$

### 2.7. Statistical Analysis to Estimate the Influence of Energy Input on Cell Growth and LA Production

A two-level two-factorial experimental design was used to determine the influences of CFV and TMP on cell growth and LA production. CFV and TMP were separately set as high-level and low-level factors (Table 1). The experiments were carried out with the orthogonal combination of CFV and TMP. Each set point was tested in triplicate. The influences of each factor were determined by analysis of variance (ANOVA) using Design Expert v8.0.6 (Stat-Ease Inc., Minneapolis, MN, USA).

## 3. Results

### 3.1. Calibration of the AFG Sensor in LA Fermentation Broth

**Figure 3:**
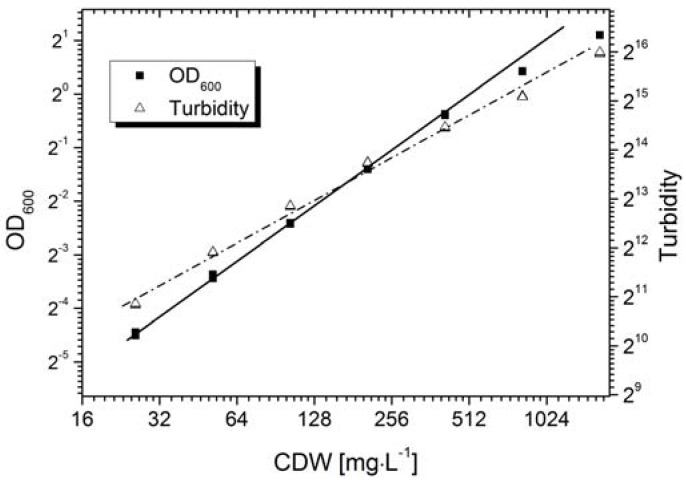
Calibration of optical density (OD_600_) and the AFG sensor in *B. coagulans* LA fermentation broth. CDW = cell dry weight. The sensor values in each sample were recorded every 10 s for a total duration of 1 min. All data are shown in the figure.

Calibration is generally required for the quantitative measurement of an objective using an optical sensor. The AFG sensor mechanism is based on the correlation of the scattering intensity and the suspended particle concentration, where the primary signal output is shown as the dimensionless “turbidity”. However, the sensor signal varies in different particle systems due to differences in particle morphology, e.g., size or shape. Figure 3 shows the calibration of the AFG sensor, and the standard off-line measurement, *i.e.*, the OD_600_ value, with fermentation broth containing *Bacillus coagulans*. The AFG sensor displayed a linear correlation (Equation (7)) with cell density up to ~0.4 g·L^−1^. A positive correlation was observed with the CDW up to ~2.0 g·L^−1^, whereas the linearity declined at a concentration higher than 0.4 g·L^−1^.

(7) $$Turbidity=53.7\cdot CDW\left(mg\right)+1178\;with\;R^{2}=0.989$$

OD measurements usually show a linear correlation with cell density when OD ≤ 0.5. We found that the OD_600_ value of the fermentation broth was proportional to the cell density up to ~0.3 g·L^−1^ (Equation (8)):

(8) $$OD_{600}=0.0019\cdot CDW\left(mg\right)\;with\;R^{2}=0.999$$

### 3.2. Online Monitoring of Biomass During Batch Fermentation

**Figure 4:**
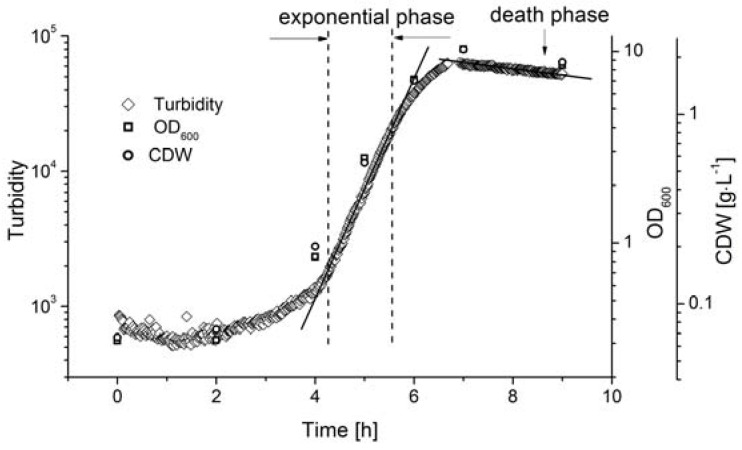
Online monitoring of biomass during the batch fermentation of *B. coagulans* for the production of LA, using optical density (OD_600_) and cell dry weight (CDW) as reference methods. The values of OD_600_ and CDW were determined three times.

For the dynamic measurement of biomass during the fermentation process, the AFG sensor was integrated into a stirred tank reactor in bypass mode, where fermentation was carried out under standard conditions (c_glu_ = 20 g·L^−1^, T = 53 °C, pH = 6.4, agitation = 150 rpm). The biomass was determined by measuring OD_600_ and by direct weighing, and was compared to the AFG sensor readout (Figure 4).

The lag-phase of *Bacillus coagulans* lasted ~3 h after inoculation. The sensor signal indicated that the biomass exponentially increased from 4.5 to 5.5 h, and the reference methods showed a similar tendency. The cell density at the end of the exponential phase did not exceed the linear range of the sensor signal, so the AFG sensor measurements could be used to calculate the maximum specific cell growth rate, as discussed below.

### 3.3. Fouling of the AFG Sensor During Batch Fermentation

**Figure 5:**
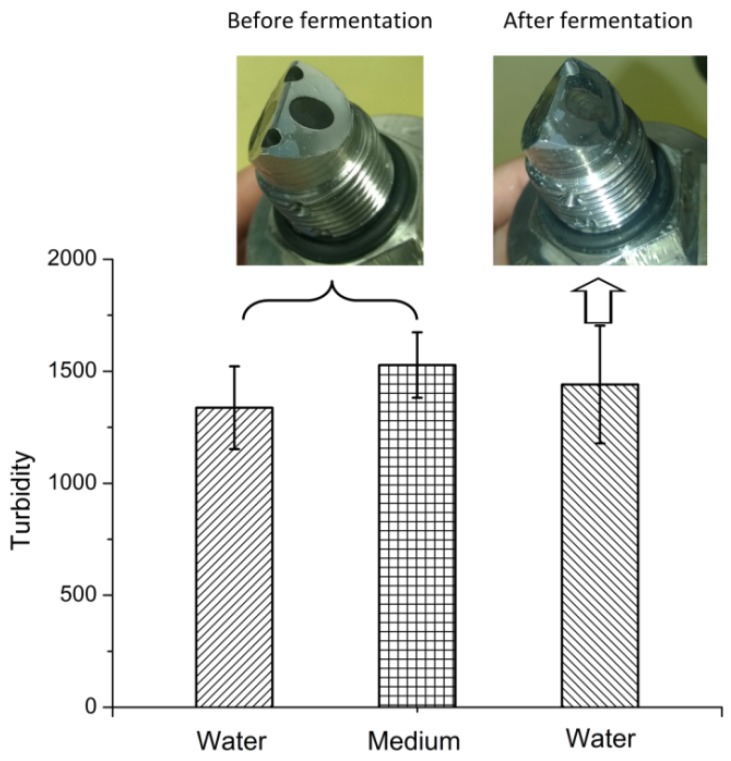
Images of the AFG sensor before and after 9 h fermentation and baseline readings taken in different media. Values are means ± standard deviation (*n* = 10) of measurements taken over 5 min.

The baseline of the AFG sensor was recorded in RO water and MRS medium before fermentation (Figure 5). Despite the brownish color of the medium, no significant change in the baseline was observed. After a 9 h fermentation, the sensor was first rinsed with RO water to remove free cells, and the baseline in water increased very slightly compared to the value before fermentation. These results imply that fouling of the optical surface is negligible.

### 3.4. Cell Growth and LA Production with Mechanical Energy Input

In an MBR system, the pump, supplying mechanical energy, causes the fermentation broth to flow tangentially through membrane tubes, and generates a pressure differential across the membrane. Membrane fouling and permeate flow during crossflow filtration can therefore be controlled by the interaction between the tangential flow and pressure differential. However, mechanical energy may also have negative effects on the vitality and growth of cells, because cells are fragile and sensitive to shear stress. High-velocity flow regimes can damage the cell membrane due to the increase in mechanical energy, causing cell death. We therefore investigated the influence of the mechanical energy input on cell growth and LA productivity. 

**Figure 6:**
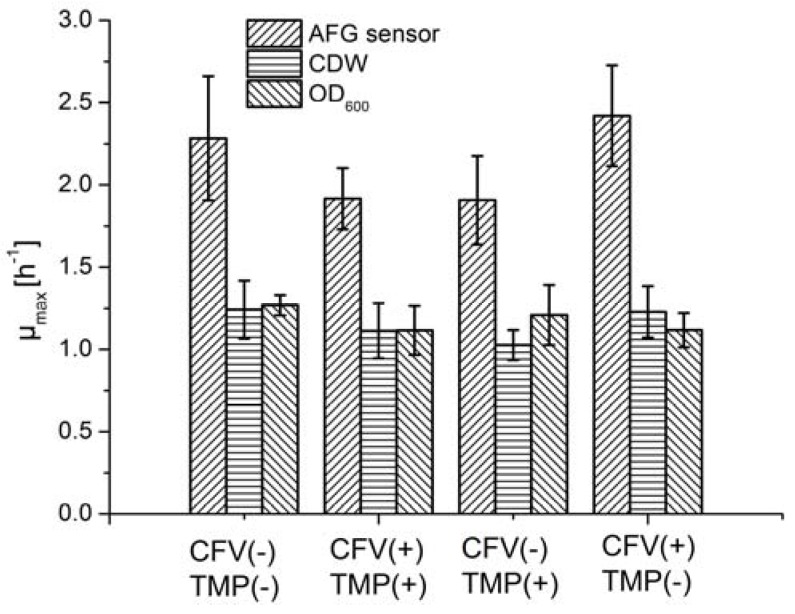
The µ_max_ of *B. coagulans* in LA batch fermentation mode, with different mechanical energy inputs, determined using the AFG sensor and offline methods. CFV: high level (+) = 1.5 m·s^−1^, low level (−) = 0.5 m·s^−1^. TMP: high level (+) = 1.2 bar, low level (−) = 0.3 bar. Values are means ± standard deviation (*n* = 3).

Fermentation was carried out by applying four conditions featuring different combinations of TMP and CFV, and the µ_max_ was determined using different methods (Figure 6). Although the average µ_max_ varied from 1.9 to 2.4 h^−1^ among the four groups when using the AFG sensor, ANOVA results suggested that neither the pressure differential nor the shear stress had a significant impact on cell growth (Table 2). The µ_max_ values estimated using offline methods fell within a narrower range than those determined with the AFG sensor. The mechanical energy input therefore did not appear to have a significant effect on the fermentation process.

### 3.5. Online Monitoring and Control of Cell Density During Filtration 

The membrane efficiency (filtration flux) in a MBR system is influenced not only by the TMP and CFV but also by the biomass concentration. In order to determine the correlation between membrane efficiency and the biomass concentration, the latter must remain constant. However, the concentration of *B. coagulans* decreases rapidly once the substrate is consumed, as shown above. Therefore, glucose solution was fed to ensure equilibrium was maintained between cell growth and cell death, and the AFG sensor was used to determine whether or not the biomass concentration remained stable over time. 

**Figure 7:**
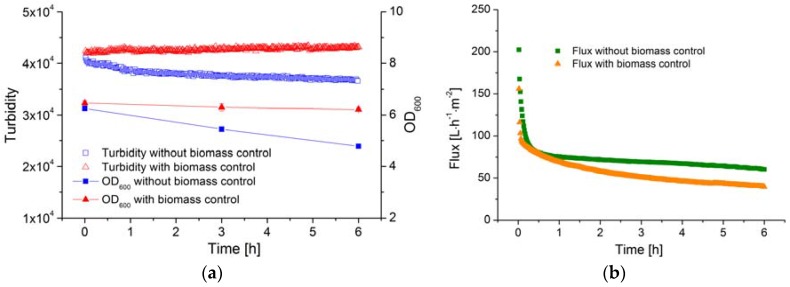
(**a**) The biomass concentration in the fermentation broth during filtration, with and without glucose feeding; OD values are means ± standard deviation (*n* = 3). (**b**) The filtration flux of the fermentation broth during filtration, with and without glucose feeding, membrane: ceramic hollow fiber membrane, TMP: 0.6 bar, CFV: 2 m·s^−1^.

The OD_600_ value and the AFG sensor signal during the filtration processes are shown in Figure 7a. Without glucose feeding, the OD_600_ of the fermentation broth declined from 6.3 to 4.8 over a period of 6 h. Over the same period, the AFG sensor signal fell from 4.1 × 10^4^ to 3.6 × 10^4^, reflecting the decline in biomass concentration, and the average specific death rate was ~0.1 h^−1^. Based on the observed biomass yield coefficient, 100 g·L^−1^ glucose solution was fed into the fermenter at a flow rate of 5 mL·h^−1^. Because the volume of the fed solution is much smaller than the total volume of fermentation broth, the latter was considered to remain constant during the 6 h filtration. The OD_600_ value and the AFG sensor signal both remained stable under these conditions, indicating that the biomass concentration had been successfully controlled and was maintained at a constant level during filtration.

The filtration flux under both conditions (with and without biomass control) is shown in Figure 7b. After filtration for 6 h, the flux in the process with biomass control was ~30% lower than in the process without biomass control. Because the filtration parameters (TMP, CFV and temperature) were identical in both experiments, the decline flux must therefore be caused by the stable biomass concentration.

## 4. Discussion

### 4.1. Characterization of the AFG Sensor

The calibration of the AFG sensor confirmed that it was suitable for the online monitoring of *B. coagulans* biomass in the LA fermentation broth. The linear correlation between the AFG sensor signal and a biomass concentration less than 0.5 g·L^−1^ can be used for the quantitative measurement of increases in cell density during the exponential phase. Despite the lack of linearity, the AFG sensor achieved a positive correlation with cell densities up to 2.2 g·L^−1^, which is comparable to other optical sensors such as an optical density probe that was used to measure cell density in *Lactobacillus casei* cultures with a linear range of up to ~1 g·L^−1^ [19]. Nonlinear correlation does not restrict further applications in quantitative biomass measurement because empirical equations can be used for correction, e.g., when using the same probe to measure the cell density of *Bacillus thuringiensis* cultures [20,21].

Another advantage of the AFG sensor is that the baseline is not affected by the color of the culture medium because NIR is less susceptible to adsorption by soluble components in the medium. In contrast, the absorption of visible light in the OD measurement is always influenced by the medium properties. These features improve the selectivity and sensitivity of the AFG sensor, which is advantageous during continuous fermentation processes because the components and color of the medium may vary with cell growth over time.

### 4.2. Online Monitoring of Cell Growth During Batch Fermentation

The AFG sensor revealed a sharper *B. coagulans* growth profile during the fermentation processes because it can gather more data in real time over shorter measurement intervals. This is advantageous for the investigation of cell growth kinetics, e.g., because the exponential phase is easier to distinguish within the complete growth profile, providing a more accurate estimate of the growth rate. The greater sensitivity of the sensor also explains why the µ_max_ estimated using the AFG sensor varies over a wider range compared to the offline methods.

Despite the better description of cell growth, the AFG sensor still has to meet a great challenge, the determination of viable cells, because the measurement of scattered light intensity cannot distinguish the living and the dead cells. However, LA production is associated with the metabolism and reproduction of cells. Hence, other measurements such as cell morphology or passive dielectric properties have to be used to determine cell viability [8,22,23,24]. Several previous investigations have used different kinds of probes to solve this problem, e.g., an *in situ* dark-field microscope has been developed to monitor cell density and viability in parallel [25]. In addition, Xiong *et al.* have applied on-line capacitance measurement to monitor viable cells in the high-cell-density cultivation of *S. cerevisiae* [26]. Druzinec *et al.* have also systematically investigated the feasibility of a multivariate impedance spectroscopy measurement to determine the amount of viable cells [27]. The viability of different types of cells, including insect cells, microbials, were successfully monitored with this approach, which is a promising candidate to complement the weakness of optical measurement and, hence, improve the accuracy and efficiency of process control.

Nevertheless, the measurement of total biomass is still important in the context of MBR systems for continuous LA production because both living and dead cells can cause membrane fouling, leading to the decrease in filtration efficiency and stability. Biomass control based on online monitoring should, therefore, be further investigated.

### 4.3. Biomass Monitoring and Control in Filtration Processes

Cells can form a cake layer on the membrane surface when the fermentation broth is filtered. This layer generates resistance during filtration, resulting in a rapid decline of flux across the membrane. The control of biomass concentration is therefore necessary in MBR systems because it not only improves substrate conversion but also influences the membrane flux [18]. The AFG sensor confirmed that the biomass concentration can be maintained at a constant level during the process we have established.

## 5. Conclusions and Outlook

Our study has confirmed that the novel AFG scattered light sensor is suitable for the online monitoring and control of LA production in a MBR system. The linear correlation between the optical signal and the biomass concentration at values up to ~0.4 g·L^−1^ allows the quantitative determination of cell growth rates using the data from the AFG sensor. The sensor behaves in a stable manner in different media without significant fouling, making it ideal for monitoring long-term LA production in a MBR system. The AFG sensor was also tested in a batch fermentation system for LA production, and was able to measure the growth of *B. coagulans* cells under conditions with different energy inputs. Our data confirmed that *B. coagulans* is a robust microorganism that is suitable for LA production in a MBR system with crossflow filtration. In addition, the AFG sensor was also successfully used during a filtration process to ensure biomass control. The correlation between filtration efficiency and biomass concentration will be investigated in more detail in future experiments.

## Figures and Tables

**Table 1:** Experimental setup showing orthogonal combinations of CFV and TMP.

Parameter	Low Level (–)	High Level (+)
CFV [m·s^−1^]	0.5	1.5
TMP [bar]	0.3	1.2

**Table 2:** The effect of mechanical energy input on cell growth, measured using different approaches. Statistical significance was determined by ANOVA.

Approach	*p* Value	Significance of Model Terms
AFG sensor	0.2700	Not significant
OD_600_	0.4948	Not significant
CDW	0.5562	Not significant

## Abbreviations

The following abbreviations are used in this manuscript:

AFG: AFGUARD^®^
ANOVA: analysis of variance
CDW: cell dry weight
CFV: crossflow velocity
LA: lactic acid
MBR: membrane bioreactor
NIR: near infrared
OD_x_: optical density at λ = x nm
TMP: transmembrane pressure
